# Two Adult Siblings With Myotonic Dystrophy Type 1 With Different Phenotypes Presenting With Chronic Respiratory Insufficiency and Sleep Apnea Syndrome

**DOI:** 10.3389/fneur.2019.00681

**Published:** 2019-07-04

**Authors:** Genta Kohno, Katsuhiko Ogawa, Masaru Kushimoto, Sho Tanaka, Jin Ikeda, Tsukasa Nishizawa, Kazutaka Mitsuke, Tomohiro Nakayama, Yutaka Suzuki, Hisamitsu Ishihara, Midori Fujishiro

**Affiliations:** ^1^Division of Diabetes and Metabolic Diseases, Department of Internal Medicine, Nihon University School of Medicine, Tokyo, Japan; ^2^Department of Internal Medicine, Nihon University Hospital, Tokyo, Japan; ^3^Division of Neurology, Department of Internal Medicine, Nihon University School of Medicine, Tokyo, Japan; ^4^Division of Nephrology, Hypertension and Endocrinology, Department of Internal Medicine, Nihon University School of Medicine, Tokyo, Japan; ^5^Division of Respiratory Medicine, Department of Internal Medicine, Nihon University School of Medicine, Tokyo, Japan; ^6^Division of Laboratory Medicine, Department of Pathology and Microbiology, Nihon University School of Medicine, Tokyo, Japan

**Keywords:** myotonic dystrophy type 1, siblings, different phenotypes, respiratory insufficiency, sleep apnea syndrome

## Abstract

Myotonic dystrophy type 1 (DM1) is an autosomal dominant disease characterized by progressive muscle weakness and myotonia along with multiple organ system involvements. Overall, DM1 patients show reduced life expectancy, mainly due to respiratory or cardiac abnormalities. Chronic respiratory impairment is associated with increased morbidity in DM1. The main ventilatory dysfunction etiology in DM1 is complex, consisting of both peripheral respiratory dysfunction and central respiratory drive dysfunction as well as upper airway muscle dysfunction leading to obstructive sleep apnea syndrome (SAS) and aspiration. Advancements in early diagnosis of DM1 and management with non-invasive therapeutic tools have improved life expectancy for DM1 patients. We present herein two siblings with DM1, a thin elder brother and an obese younger sister with visceral fat accumulation. Although neither had voluntary symptoms related to respiratory dysfunction, their apnea-hypopnea indices revealed severe SAS and subsequent arterial blood gases studies showed hypercapnia as well as hypoxia, suggesting central nervous system involvement with peripheral respiratory dysfunction. Non-invasive positive pressure ventilation during sleep was started following pulmonary assessment. Respiratory function should be assessed in DM1 patients, even those free of respiratory symptoms, because respiratory muscle weakness occurs in a high percentage of these patients and will shorten their lives.

## Introduction

Myotonic dystrophy type 1 (DM1) is the most common type of muscular dystrophy in adults, caused by a trinucleotide cytosine-thymine-guanine (CTG) repeat expansion in the dystrophia myotonica protein kinase (*DMPK*) gene on chromosome 19q13.3, and represents multiple organ symptoms including respiratory dysfunction (1). As a cause of respiratory dysfunction in DM1, besides a restrictive ventilatory pattern due to respiratory muscle weakness, involvement of the central nervous system (CNS) has recently been reported (2–5). Sleep apnea syndrome (SAS) is a prevalent disorder particularly among obese patients including those with metabolic syndrome (MetS) which is associated with risks of cardiovascular morbidity and mortality, type 2 diabetes, and all-cause mortality and are also reportedly associated with DM1, i.e., 63.6% of DM1 patients who underwent polysomnography had obstructive SAS due to weakness of the expiratory muscles, or hemidiaphragm palsy secondary to phrenic nerve dysfunction (3–7). We present herein siblings with DM1, an older brother and a younger sister with severe SAS accompanied by hypercapnia, suggesting that both probably had central respiratory dysfunction, although no voluntary symptoms were noted.

## Case Report

### Case 1 (A 44-Year-Old Male)

**Figure 1:**
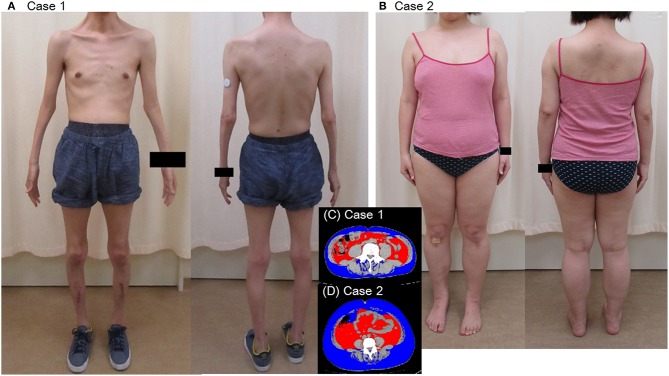
Appearance **(A,B)** and visceral fat area on CT of the patients **(C,D)**.

**Figure 2:**
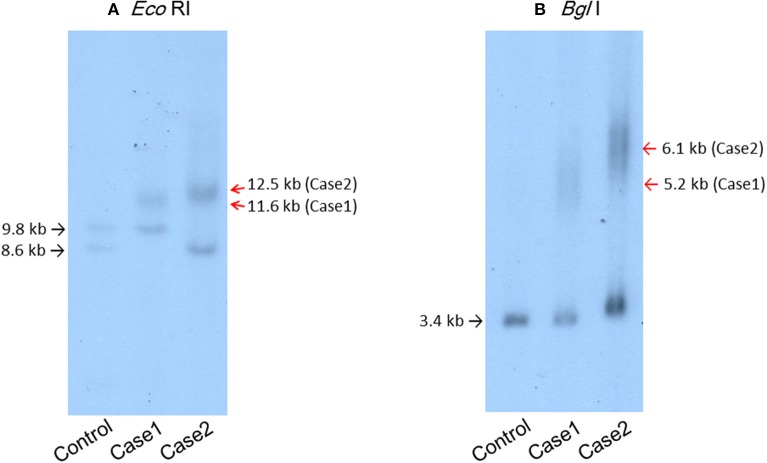
Southern blot analysis of the patients' genomic DNA samples digested with *Eco*RI **(A)** or *Bgl*I **(B)** and then probed with the *Bam*HI fragment containing the *DMPK* CTG repeat region. **(A)** Lane 1, genomic DNA from a control individual with an *Eco*RI polymorphism (10) in one allele. Lane 2, case 1. Lane 3, case 2.

A 44-year-old Japanese male, an office worker, was transported to our emergency department with a complaint of temporary loss of consciousness. He had experienced fatigue for 3 years and had fallen easily in his daily life starting 2 months before admission. He was feverish and had a cough and phlegm for several days before admission. On the way to work, he felt drowsy and fell down the stairs of the train station, necessitating transport to our hospital by ambulance. The peripheral blood analysis noted mild elevation of white blood cells. The blood chemistry test disclosed mild elevation of C-reactive protein and mild liver dysfunction (Table 1A). He was thin, i.e., his height was 174 cm and he weighed 52 kg (Figure 1). Chest computed tomography (CT) showed severe infiltration in the upper posterior fields of both lungs as well as food debris in the esophagus. He was diagnosed as having aspiration pneumonia. His past medical history included diabetes mellitus that had been treated by a local doctor; glycated hemoglobin was approximately 7%. He had experienced ileus six times since age 30 years. He was not married. His father had died of dilated cardiomyopathy at the age of 70 and had also been thin. On the other hand, his mother was healthy but his maternal uncle was diabetic. His younger sister (Case 2) also had mild muscle weakness of the four extremities. Aspiration pneumonia was treated by intravenous administration of antibiotics under conditions of food-take restriction combined with temporary insulin infusion, and the respiratory symptoms subsided. On the neurological examinations after improvement of pneumonia, he presented with typical clinical manifestations of DM1 (8), such as forehead balding, hatchet face with bilateral ptosis, nasal speech, mild muscle weakness of the four extremities, handgrip myotonia, and diffuse muscle atrophy. Electromyography performed to test the biceps brachii muscle and femoral quadriceps muscle on the left side revealed frequent myotonic discharges. Because DM1 was strongly suspected, we recommended that, after providing informed consent during genetic counseling, he undergo genetic testing for DM1 together with his younger sister. The number of CTG repeats in the *DMPK* gene was abnormally expanded to about 600 repeats in the elder brother and about 900 repeats in the younger sister, in contrast to those of healthy individuals who have 5 to 37 repeats (9), thereby confirming the diagnosis of DM1 in these siblings (Figure 2).

**Table 1:** Laboratory values and arterial blood gas of Case 1.

	**a**	**b**
**(A) LABORATORY VALUES OF CASE 1**		
WBC (/l)	6,400	6,100
RBC (× 10^4^/μl)	389	465
Hb (g/dl)	12.3	14.1
Plt (× 10^4^/μl)	13	19
CRP (mg/dl)	**8.9**	0.1
Total protein (g/dl)	**5.7**	6.9
Total bilirubin (mg/dl)	1.3	0.6
AST (GOT) (U/l)	**243**	24
ALT (GPT) (U/l)	**202**	21
Alkaline phosphatase (U/l)	**579**	293
Gamma-glutamyl transferase (U/l)	**193**	71
Creatine kinase (U/l)	160	191
Blood urea nitrogen (mg/dl)	8.2	14.4
Creatinine (mg/dl)	**0.5**	**0.6**
Sodium (mmol/l)	142	146
Potassium (mmol/l)	4.1	4.9
Chloride (mmol/l)	104	104
Uric acid (mg/dl)	4.2	4.8
Total cholesterol (mg/dl)	189	204
HDL-C (mg/dl)	65	77
Triglyceride (mg/dl)	64	145
Glucose (mg/dl)	**146**	**113**
Glycated hemoglobin (%)	**7.0**	**7.3**
**(B) ARTERIAL BLOOD GAS VALUES OF CASE 1**		
pH	7.4	7.4
PaCO_2_ (mmHg)	44.0	**48.3**
PaO_2_ (mmHg)	**170.9**	81.5
HC${\text{O}}_{3}^{-}$ (mmol/l)	25.3	**29.2**
BE (mmol/l)	−0.5	**3.6**
SaO_2_ (%)	99	96

*Numbers in bold type lie outside of the reference range*.

*a, Data obtained on admission to an emergency department with 10 L/min of oxygen from a mask with a reservoir*.

*b, Data obtained at outpatient care 3 months after discharge, before starting NPPV*.

*ALT, alanine aminotransferase; AST, aspartate aminotransferase; CRP, C-reactive protein; HDL-C, high-density lipoprotein cholesterol*.

### Case 2 (A 41-Year-Old Woman)

His younger sister was a 41-year-old woman, a housewife. She was obese according to the Japanese criteria (11), i.e., she was 156 cm in height, weighing 69.6 kg (102 cm waist circumference and a 164.2 cm^2^ visceral fat area calculated employing abdominal CT). Slightly impaired fasting glucose levels had been pointed out on her medical check-ups since 3 years earlier, but she had never consulted a doctor. She had noted repeated falls and injuries for the past 5–6 years. She had married at the age of 37 and had undergone infertility treatment. When she was admitted for detailed examination to our hospital, she showed distal muscle weakness of the four extremities as described above, handgrip myotonia, and distal muscle atrophy, which were consistent with the characteristics of DM1. The laboratory data showed elevated fasting glucose with dyslipidemia, which met the Japanese criteria for metabolic syndrome (MetS) (Table 2A) (12), and an oral glucose tolerance test using 75 gram glucose showed impaired glucose tolerance (IGT) (Table 2B), followed by starting treatment with diet and exercise. Based on the presence of MetS, a sleep study was conducted using an apnomonitor Type 4 (SAS-2100, NIHON KOHDEN, Tokyo, Japan) (Table 3) (6, 7, 13). The apnea-hypopnea index (AHI) was defined as the average number of apnea and hypopnea episodes per hour of recording time in bed. The younger sister's AHI was high at 55.9, which suggested that she had severe SAS, as defined by an AHI of 30 or more, though no snoring, apneic episodes, nocturnal dyspnea or signs of daytime somnolence were observed (14, 15). Her arterial blood gas (ABG) showed hypoxia accompanied by hypercapnia and her lung function test, using spirometry (CHESTAC-8900, Chest, Tokyo, Japan), revealed a decreased percent predicted vital capacity (VC) of 67.9% while Forced Expiratory Volume in 1 s (FEV1)/ Forced Vital Capacity (FVC) (Gaensler index) was adequate at 87.9% (Table 3) (16).

Table 2Laboratory values of Case 2.

**Table:** 

**(A) LABORATORY VALUES OF CASE 2 OBTAINED ON ADMISSION**		
WBC (/l)	7,100
RBC (× 10^4^/ml)	465
Hb (g/dl)	12.6
Plt (× 10^4^/ml)	19
AST (GOT) (U/l)	32
ALT (GPT) (U/l)	37
Alkaline phosphatase (U/l)	**399**
Gamma-glutamyl transferase (U/l)	**91**
Creatine kinase (U/l)	160
Blood urea nitrogen (mg/dl)	11.1
Creatinine (mg/dl)	**0.4**
Sodium (mmol/l)	146
Potassium (mmol/l)	4.5
Chloride (mmol/l)	109
Uric acid (mg/dl)	3.7
Total cholesterol (mg/dl)	**254**
HDL-C (mg/dl)	60
Triglyceride (mg/dl)	**188**
Glucose (mg/dl)	129
Glycated Hemoglobin (%)	6.2

**Table:** 

**(B) DATA OBTAINED BY 75 GRAM OGTT TEST THE DAY AFTER ADMISSION OF CASE 2**		
	**Glucose (mg/dl)**	**Insulin (mU/ml)**
Fasting	90	6.5
OGTT Post-30 min	151	65.6
OGTT Post-60 min	162	50.3
OGTT Post-90 min	175	47.9
OGTT Post-120 min	176	56.9

*Numbers in bold type lie outside of the reference range*.

*ALT, alanine aminotransferase; AST, aspartate aminotransferase; HDL-C, high-density lipoprotein cholesterol*.

*OGTT, oral glucose tolerance test*.

**Table 3:** Summary of respiratory function examinations of Case 1 and Case 2.

	**Case1**	**Case2**
**(A) APNOMONITOR TYPE 4**		
AHI (events/h of sleep)	**55.1**	**55.9**
Apnea index (events/h of sleep)	**33.9**	**27.4**
Hypopnea index (events/h of sleep)	**21.2**	**28.5**
3% Oxygen desaturation index (events/h of sleep)	**40.7**	**54.2**
Minimum nocturnal oxygen saturation (%)	**67**	**63**
Mean nocturnal oxygen saturation (%)	**93**	**87**
Maximum apnea duration (s)	**101.7**	**101.8**
Mean apnea duration (s)	**32.5**	**20.1**
**(B) SPIROMETRY**		
direct VC (L) value	2.05	1.88
VC (% pred)	**51.9**	**67.9**
direct FVC (L) value	2.12	1.82
FVC (% pred)	**53.7**	**65.7**
direct FEV1 (L) value	1.99	1.6
FEV1 (% pred)	**56.2**	**62.7**
FEV1% (Gaensler index; FEV1/FVC)	93.9	87.9
**(C) ARTERIAL BLOOD GAS**		
pH	7.4	**7.4**
PaCO_2_ (mmHg)	**48.3**	**48.5**
PaO_2_ (mmHg)	81.5	**71.7**
HC${\text{O}}_{3}^{-}$ (mmol/l)	**29.2**	**27.8**
BE (mmol/l)	**3.6**	2.0
SaO_2_ (%)	96	**94.0**

Numbers in bold type lie outside of the reference range.

*AHI, apnea-hypopnea index; FEV1, forced expiratory volume in 1 s; FVC, forced vital capacity; VC, vital capacity; pred, predicted*.

We then also analyzed the respiratory function of the elder brother (Case 1), although, like his sister, he had not complained of voluntary symptoms suggestive of SAS. His AHI examined using an apnomonitor Type 4 was 55.1 and his ABG showed CO_2_ retention without hypoxemia (Table 1B). His percent predicted VC was decreased, at 51.9%, while the Gaensler index was sufficient at 93.9%, similar to his sister's values (Table 3). His aspiration pneumonia was regarded as possibly being due to DM1, which can cause expiratory muscle weakness. The indications for non-invasive positive pressure ventilation (NPPV) were assessed by an experienced pulmonologist. Based on chronic alveolar hypoventilation (patients with decreased percent predicted VC are at high risk), diurnal hypercapnia (PaCO_2_ ≥ 45 mmHg) according to the ABG, evidence of nocturnal hypoventilation on nocturnal pulse-oximetry, defined as SpO_2_ < 92% more than 4 times during the entire sleep time or >4% of the entire sleep time as well as AHI≥10 events/h, these two siblings were started on NPPV. The NPPV consisted of 6 cmH_2_O of inspiratory positive airway pressure, 4 cmH_2_O of expiratory positive airway pressure and a 12 bpm back-up rate in spontaneous/timed mode during sleep (4, 5, 16–18). These two siblings have been followed at outpatient care by the same pulmonologist and there has been no apparent worsening of symptoms since starting NPPV therapy. Both patients have shown good compliance. The ABG of the brother revealed slight elevation of PaCO_2_, that is, pH 7.4, PaCO_2_ 51.5 mmHg, PaO_2_ 76.9 mmHg, HC${\text{O}}_{3}^{-}$ 28.5 mmol/l, BE 2.1 mmol/l, and SaO_2_ 95%. Because the sister's arteries are very thin, making it difficult for us to determine her ABG, spirometry was performed 3 months after initiating NPPV therapy and revealed almost no interval changes as compared to the baseline data before starting NPPV, with the direct value of VC being 1.9 L, VC 68.3% pred, direct value of FVC 1.84 L, FVC 66.2% pred, direct value of FEV1 1.52 L, FEV1 59.4% pred, and FEV1% (Gaensler index; FEV1/FVC) 101.8%.

As to other complications, the brother showed negative results on brain magnetic resonance imaging (MRI), echocardiography, abdominal ultrasound and, except for the diabetes, on endocrinologic examinations (8). The sister underwent brain MRI which showed localized white matter hyperintense lesions in the right temporal lobe, a common finding in DM1 (19), which may have accounted for her hypoventilation (5). Other studies including echocardiography, abdominal ultrasound, and endocrinological examinations yielded essentially normal results except for cataracts and IGT.

## Discussion

Patients with DM1 typically present with distally predominant muscular atrophy often resulting in an emaciated appearance. At the same time, MetS is significantly more common in DM1 patients than in the general population, which is considered to be due to a sedentary lifestyle related to muscle weakness (20). As mentioned above, body types differed markedly between our DM1 siblings, though both had respiratory dysfunction. Our Case 2 presented with MetS, prompting us to investigate her for sleep disorders. These investigations yielded evidence suggestive of severe SAS, while ABG and lung function tests detected type 2 respiratory failure, indicating that the main cause of her respiratory disorder was likely to be DM1 rather than the concomitant MetS (5, 6).

The DM1 phenotype is known to show extremely wide variability among affected patients, with some being asymptomatic while others have severe congenital manifestations (1, 8, 9). Among these symptoms and features, respiratory muscle weakness occurs in a high percentage of patients with DM1 in an early stage of the disease, i.e., the overall prevalence of ventilatory restriction is reportedly 36% (2, 21), and chronic respiratory failure can develop during its clinical course (5, 22, 23). Respiratory dysfunction in DM1 is predominantly a restrictive ventilatory pattern associated with alveolar hypoventilation, chronic hypercapnia, and sleep disturbance in the form of sleep apnea and sleep-related disordered breathing. Central respiratory drive is known to be significantly involved in the alveolar hypoventilation in DM1 (2–5), but the precise mechanism in the CNS has yet to be elucidated. Furthermore, there is no consensus in the literature regarding the relationship between CTG repeat length and the severity of respiratory dysfunction (3, 5). Sleep apnea may increase morbidity and mortality by leading to daytime respiratory failure, with expiratory muscle dysfunction often manifesting sooner than that of inspiratory muscles, possibly leading to early recurrent pneumonia due to a weak cough and insufficient airway clearance (5, 8). Despite restrictive lung syndrome progressing slowly (24), problems and complications related to the respiratory system still represent the main cause of reduced life expectancy in DM1, i.e., respiratory problems are reportedly among the leading causes of death in adult DM1 patients (51–76%) with most of these patients dying between 50 and 60 years of age (5, 25). Furthermore, since DM1 affects the CNS, studies have pointed out that an abnormal sensitivity of the central respiratory drive to chemical blood changes, particularly of [CO_2_], might contribute to the pathogenesis of respiratory dysfunction in DM1 (2, 26). Because we could not examine the causes of chronic respiratory insufficiency of our patients in detail, we can rule out neither respiratory muscle weakness nor CNS involvement as potential causes of respiratory insufficiency. Furthermore, DM1, though not necessarily by itself, can contribute, along with other coexisting sleep breathing disorders, to the conditions of patients like ours. However, we consider the main cause to be DM1 in our cases, based on the results of whole body screening examinations showing no signs of coexisting sleep breathing disorders such as obstructive pulmonary diseases, emphysematous lung lesions or intestinal lesions suggestive of restrictive lung diseases.

The development of chronic, progressive respiratory failure can have prognostic effects, particularly in terms of survival, in many neuromuscular diseases including DM1. Early detection of respiratory dysfunction in addition to early diagnosis of DM1 is crucial. Therapeutic management employing non-invasive therapeutic tools such as NPPV may increase the life expectancies of DM1 patients (3, 4).

## Conclusion

Respiratory system function should be examined in DM1 patients, even those with minimal or no symptoms, because respiratory muscle weakness occurs in a high percentage of DM1 patients, and chronic respiratory failure may develop (5, 22, 23).

## Data Availability

All datasets generated and analyzed for this study are included in the manuscript and/or the supplementary files.

## Ethics Statement

Both subjects gave written informed consent for publication of the information and images related to this case report. As this is a case report without experimental intervention, no formal research ethics approval was required.

## Author Contributions

GK, MK, ST, JI, TNi, and KM substantially contributed to the acquisition, analysis, or interpretation of the data obtained. KO, HI, and MF drafted the manuscript or revised it critically for important intellectual content. TNa and YS provided approval for publication of the content. All of the authors agreed to be accountable for all aspects of this study in ensuring that questions related to the accuracy or integrity of any part of the work are appropriately investigated and resolved.

### Conflict of Interest Statement

The authors declare that the research was conducted in the absence of any commercial or financial relationships that could be construed as a potential conflict of interest.

## Abbreviations

ABG: arterial blood gas
AHI: apnea-hypopnea index
CNS: central nervous system
CT: computed tomography
CTG: cytosine-thymine-guanine
DM1: myotonic dystrophy type 1
DMPK: dystrophia myotonica protein kinase
IGT: impaired glucose tolerance
MRI: magnetic resonance imaging
NIV: non-invasive therapeutic tools
NPPV: non-invasive positive pressure ventilation
SAS: sleep apnea syndrome.

## References

[1] Meola G Cardani R. Myotonic dystrophies: an update on clinical aspects, genetic, pathology, and molecular pathomechanisms. Biochim Biophys Acta. (2015) 1852:594–606. 10.1016/j.bbadis.2014.05.019 24882752

[2] Poussel M Thil C Kaminsky P Mercy M Gomez E Chaouat A. Lack of correlation between the ventilatory response to co2 and lung function impairment in myotonic dystrophy patients: evidence for a dysregulation at central level. Neuromuscul Disord. (2015) 25:403–8. 10.1016/j.nmd.2015.02.006 25753091

[3] Hawkins AM Hawkins CL Abdul Razak K Khoo TK Tran K Jackson RV. Respiratory dysfunction in myotonic dystrophy type 1: a systematic review. Neuromuscul Disord. (2018) 29:198–212. 10.1016/j.nmd.2018.12.002 30765255

[4] Rossi S Della Marca G Ricci M Perna A Nicoletti TF Brunetti V. Prevalence and predictor factors of respiratory impairment in a large cohort of patients with myotonic dystrophy type 1 (dm1): a retrospective, cross sectional study. J Neurol Sci. (2019) 399:118–24. 10.1016/j.jns.2019.02.012 30798109

[5] Sansone VA Gagnon C. 207th enmc workshop on chronic respiratory insufficiency in myotonic dystrophies: management and implications for research. Neuromuscul Disord. (2015) 25:432–42. 10.1016/j.nmd.2015.01.011 25728518

[6] Gaines J Vgontzas AN Fernandez-Mendoza J Bixler EO. Obstructive sleep apnea and the metabolic syndrome: the road to clinically-meaningful phenotyping, improved prognosis, and personalized treatment. Sleep Med Rev. (2018) 42:211–9. 10.1016/j.smrv.2018.08.009 30279095 PMC6221996

[7] Vgontzas AN Bixler EO Chrousos GP. Sleep apnea is a manifestation of the metabolic syndrome. Sleep Med Rev. (2005) 9:211–24. 10.1016/j.smrv.2005.01.006 15893251

[8] Wenninger S MF Schoser B. Core clinical phenotypes in myotonic dystrophies. Front Neurol. (2018) 9:303. 10.3389/fneur.2018.00303 29770119 PMC5941986

[9] Valaperta R Sansone V Lombardi F Verdelli C Colombo A Valisi M. Identification and characterization of dm1 patients by a new diagnostic certified assay: neuromuscular and cardiac assessments. Biomed Res Int. (2013) 2013:958510. 10.1155/2013/958510 23762868 PMC3665172

[10] Shelbourne P Davies J Buxton J Anvret M Blennow E Bonduelle M. Direct diagnosis of myotonic dystrophy with a disease-specific DNA marker. N Engl J Med. (1993) 328:471–5. 10.1056/nejm199302183280704 8421476

[11] Examination Committee of Criteria for 'Obesity Disease' in Japan Japan Society for the Study of Obesity New criteria for 'obesity disease' in Japan. Circ J. (2002) 66:987–92. 12419927 10.1253/circj.66.987

[12] [definition and the diagnostic standard for metabolic syndrome–committee to evaluate diagnostic standards for metabolic syndrome] Nihon Naika Gakkai Zasshi. (2005) 94:794–809. 15865013

[13] Kiyokuni M Kawashima C Konishi M Sakamaki K Iwata K Nakayama N. Relationship between sleep-disordered breathing and renal dysfunction in acute coronary syndrome. J Cardiol. (2018) 71:168–73. 10.1016/j.jjcc.2017.07.017 29249245

[14] Chesson AL Jr Berry RB Pack A American Academy of Sleep Medicine, American Thoracic Society, American College of Chest Physicians. Practice parameters for the use of portable monitoring devices in the investigation of suspectedobstructive sleep apnea in adults. Sleep. (2003) 26:907–13. 10.1093/sleep/26.7.907 14655928

[15] Marin JM CS Vicente E Agusti AG Long-term cardiovascular outcomes in men with obstructive sleep apnoea-hypopnoea with or without treatment with continuous positive airway pressure: an observational study. Lancet. (2005) 365:1046–53. 10.1016/S0140-6736(05)71141-7 15781100

[16] Miller MR Hankinson J Brusasco V Burgos F Casaburi R Coates A. Standardisation of spirometry. Eur Respir J. (2005) 26:319–38. 10.1183/09031936.05.00034805 16055882

[17] Birnkrant DJ Bushby K Bann CM Alman BA Apkon SD Blackwell A. Diagnosis and management of duchenne muscular dystrophy, part 2: respiratory, cardiac, bone health, and orthopaedic management. Lancet Neurol. (2018) 17:347–61. 10.1016/s1474-4422(18)30025-5 29395990 PMC5889091

[18] Monteiro R Birnkrant DJ Gonçalves MR Pinto T Winck JC. Genetics correlates with lung function and nocturnal ventilation in myotonic dystrophy. Sleep Breath. (2013) 17:1087–92. 10.1007/s11325-013-0807-6 23319325

[19] Censori B Provinciali L Danni M Chiaramoni L Maricotti M Foschi N. Brain involvement in myotonic dystrophy: MRI features and their relationship to clinical and cognitive conditions. Acta Neurol Scand. (1994) 90:211–7. 7847063 10.1111/j.1600-0404.1994.tb02708.x

[20] Vujnic M Peric S Popovic S Raseta N Ralic V Dobricic V. Metabolic syndrome in patients with myotonic dystrophy type 1. Muscle Nerve. (2015) 52:273–7. 10.1002/mus.24540 25487787

[21] Poussel M Kaminsky P Renaud P Laroppe J Pruna L Chenuel B. Supine changes in lung function correlate with chronic respiratory failure in myotonic dystrophy patients. Respir Physiol Neurobiol. (2014) 193:43–51. 10.1016/j.resp.2014.01.006 24440340

[22] Reardon W Newcombe R Fenton I Sibert J Harper PS. The natural history of congenital myotonic dystrophy: mortality and long term clinical aspects. Arch Dis Child. (1993) 68:177–81. 10.1136/adc.68.2.177 8481038 PMC1029229

[23] Boentert M Wenninger S Sansone VA. Respiratory involvement in neuromuscular disorders. Curr Opin Neurol. (2017) 30:529–37. 10.1097/WCO.0000000000000470 28562381

[24] Thil C Agrinier N Chenuel B Poussel M. Longitudinal course of lung function in myotonic dystrophy type 1. Muscle Nerve. (2017) 56:816–8. 10.1002/mus.25604 28181267

[25] Mathieu J Allard P Potvin L Prevost C Begin P. A 10-year study of mortality in a cohort of patients with myotonic dystrophy. Neurology. (1999) 52:1658–62. 10331695 10.1212/wnl.52.8.1658

[26] Niedermeyer S Murn M Choi PJ. Respiratory failure in amyotrophic lateral sclerosis. Chest. (2019) 155:401–8. 10.1016/j.chest.2018.06.035 29990478

